# Yolk and Casein Sequence Self-Assembly for Low-Oil Emulsion Gel and Its Application in Low-Fat Mayonnaise

**DOI:** 10.3390/foods14010036

**Published:** 2024-12-26

**Authors:** Anqi Bi, Beiwei Zhu, Ning Cong, Ming Du, Chao Wu, Ling Zhang, Yan Guo, Tingting Cheng, Pei Yu, Xianbing Xu

**Affiliations:** 1 National Engineering Research Center of Seafood, Collaborative Innovation Center of Provincial and Ministerial Co-construction for Seafood Deep Processing, School of Food Science and Technology, Dalian Polytechnic University, Dalian 116034, China; bianqi96@163.com (A.B.); zhubeiwei@163.com (B.Z.); 18340129345@163.com (N.C.); duming@dlpu.edu.cn (M.D.); wuchao@dlpu.edu.cn (C.W.); zhanglingyyxs@163.com (L.Z.); 2 Dalian Kowa Foods Co., Ltd., Dalian 116034, China; guoyan995@163.com (Y.G.); chenti12@163.com (T.C.); 3 College of Food Science and Engineering, Dalian Ocean University, Dalian 116023, China; yupei@dlou.edu.cn

**Keywords:** low-fat mayonnaise, yolk, casein, post-acid treatment

## Abstract

High dietary fat food such as mayonnaise (70–80% oil content) can induce obesity and cardiovascular diseases, thus reducing their oil content is required. However, the development of low-fat mayonnaise is still a big challenge since reducing oil content will increase the fluidity, induce phase separation and decrease the stability of mayonnaise. Herein, we provide a novel strategy for developing yolk–casein-based low-fat mayonnaise (30% oil content) with a similar texture to commercial high-fat mayonnaise through post-acidification. Unexpectedly, compared with pre-acid-treated low-fat mayonnaise, the G′ and viscosity of the post-acid-treated low-fat mayonnaise were significantly improved by 77.80% and 90.18%, respectively. The semisolid properties required for low-fat mayonnaise were realized by forming a dense yolk–casein self-assembly network structure. This study provides a novel perspective for constructing edible soft-solid products with low fat intake.

## 1. Introduction

Mayonnaise, as a semisolid oil-in-water emulsion condiment, has been widely consumed by more than 85% of the population in the world due to its creamy mouthfeel and unique flavor [1,2,3]. However, traditional mayonnaise, with about 70–80% fat, is a typical high-fat product [2,4]. It is reported that overconsumption of high-fat foods increases the risk of several chronic diseases such as obesity and cardiovascular diseases [4,5]. Therefore, low-fat mayonnaise is required for healthy food development.

Generally, the application of protein-based fat replacers is considered an excellent strategy for preparing low-fat mayonnaise with a similar texture to commercial high-fat mayonnaise [6,7]. Although egg white protein and whey protein are available for the preparation of low-fat mayonnaise [2,6], the addition of thickeners such as pectin, flaxseed gum, acacia gum, and xanthan gum is essential to increase the viscosity and reduce the fluidity of the products during the preparation of low-fat mayonnaise [2,6,8]. However, almost all thickeners mentioned for the preparation of low-fat mayonnaise are chemically modified [9], thus deteriorating the consumer acceptance of the products. Therefore, a novel strategy for preparing thickener-free low-fat mayonnaise with clean-label is urgently required.

Recently, developing protein-based fat replacers for preparing low-fat mayonnaise has increased in interest. Casein is an amphiphilic self-assembling protein with natural biocompatibility, low-cost, readily accessible, and excellent emulsifying advantages, which has been widely studied as an effective emulsion stabilizer [10,11,12]. To date, casein has been widely applied in various dairy products, such as cheese and yogurt [13,14]; thus, casein has great potential for preparing mayonnaise. However, for low-fat mayonnaise, typical acidic seasoning, the impact of fat replacement by casein on the stability and texture of emulsions under acidic conditions is still unclear.

This study provides a simple strategy for developing low-fat mayonnaise (30% oil content) via casein and yolk sequence self-assembly by post-acid treatment. The interfacial properties, morphology, and rheological properties of the resultant low-fat mayonnaise are studied. Moreover, a comparison is made between the developed low-fat mayonnaise and commercial high-fat mayonnaise in terms of their sensory characteristics. To our knowledge, this is the first report on developing thickener-free protein-based low-fat mayonnaise.

## 2. Materials and Methods

### 2.1. Materials

Casein and fluorescein isothiocyanate (FITC) were sourced from Aladdin Biochemical Technology Co., Ltd. (Shanghai, China). The Nile Red was provided by Sangon Biotech (Shanghai) Co., Ltd. (Shanghai, China). The liquid yolk was provided by Dalian Kowa Foods Co., Ltd. (Dalian, China). The soybean oil and Kewpie mayonnaise were purchased from Jing Dong Supermarket. The Na_2_CO_3_, NaHCO_3_, DMSO, KOH, and HCl used were all of reagent grade.

### 2.2. Methods

#### 2.2.1. Preparation of Yolk–Casein Solution

The 10% (*w*/*v*) casein solution was adjusted to pH 11. Then, the liquid yolk and resultant casein solution were mixed at mass ratios of 1:1, 1:2, and 1:3, resulting in yolk–casein solutions.

#### 2.2.2. Interfacial Tension

The interfacial properties of the emulsions were determined using a drop shape analyzer (DSA 25, KRÜSS, Hamburg, Germany). The Interfacial tension was measured according to the methods reported by Xiao et al. [15] with slight modifications.

The interfacial tension of the pre-acid-treated group was obtained as follows: 7 μL of the yolk–casein solution with a mass ratio of 1:3 (pH 4) was directly excluded and suspended in the cuvette filled with soybean oil. Advanced software (KRÜSS, Germany) was further applied to record the interfacial tension.

For the post-acid-treated group, a drop of non-acid-modified solution (pH 11 casein solution, yolk–water solution with a mass ratio of 1:3, and the prepared yolk–casein solution with a mass ratio of 1:3 in Method 2.2.1) was suspended in a cuvette filled with soybean oil, respectively. Then, 1 mol L^−1^ HCl was injected into the suspending droplet, respectively. Notably, the total volume of all droplets with 1 mol L^−1^ HCl was 7 μL. The extrusive droplet volume and the added volume of acid were based on their volume in the pre-acid-treated group to ensure that the pH of all solutions was consistent with that of the pre-acid-treated group (pH 4). Advanced software (KRÜSS, Germany) was further applied to record the interfacial tension.

For the non-acid-treated group, 7 μL of yolk–casein solution with a mass ratio of 1:3 in Method 2.2.1 without acid modification was directly excluded and suspended in the cuvette filled with soybean oil. Advanced software (KRÜSS, Germany) was further applied to record the interfacial tension.

#### 2.2.3. Preparation of Low-Fat Mayonnaise

The pre-acid-treated low-fat mayonnaises were obtained as follows: First, the prepared pH 11 casein solution, yolk–water solutions (with mass ratios of 1:1, 1:2, and 1:3), and yolk–casein solutions (with mass ratios of 1:1, 1:2, and 1:3) in Method 2.2.1 were adjusted to pH 4 with 12 mol L^−1^ HCl, respectively. Then, soybean oil (φ = 30%) was added slowly to the pH 4 yolk–casein solution using a T25 IKA homogenizer (IKA^®^ Werke, Staufen, Germany) at a shear rate of 3500 rpm. Thereby, pre-acid-treated low-fat mayonnaises were obtained.

The post-acid-treated low-fat mayonnaises were obtained as follows: Soybean oil (φ = 30%) was added slowly to the pH 11 casein solution (10% (*w*/*v*)), yolk–water solutions (with mass ratios of 1:1, 1:2, and 1:3), and the prepared yolk–casein solutions (with mass ratios of 1:1, 1:2, and 1:3) in Method 2.2.1 using a homogenizer at 3500 rpm, respectively, resulting the non-acid-treated low-fat mayonnaises. Subsequently, 12 mol L^−1^ HCl was added to the non-acid-treated low-fat mayonnaises and the pH of the low-fat mayonnaises was adjusted to 4. Thereby, post-acid-treated low-fat mayonnaises were obtained.

#### 2.2.4. Preparation of Yolk–Casein-Mixture-Based Low-Fat Mayonnaise with Stained Yolk

A total of 4.5 g liquid yolk was complexed with 50 mL fresh carbonate buffer (50 mM, pH 9) to obtain yolk dilution. Then, 2.5 mL FITC dissolved in anhydrous DMSO at 1 mg/mL concentration was added to the yolk dilution. The dyed yolk dilution was stirred at 4 °C under dark conditions for 8 h. The free FITC was removed by dialysis for 24 h. Then, these samples were lyophilized to obtain the dyed yolk. Next, the lyophilized dyed yolk was complexed with water to recover the original mass of 4.5 g liquid yolk, obtaining dyed liquid yolk. Then, the dyed liquid yolk was complexed with 10% (*w*/*v*) casein solution according to Method 2.2.1, acquiring casein–dyed yolk solution. Finally, the low-fat mayonnaises with pre-acid and post-acid treatment were prepared following Method 2.2.3 to obtain the stained yolk–casein-mixture-based low-fat mayonnaise with pre-acid and post-acid treatment.

#### 2.2.5. Confocal Laser Scanning Microscopy (CLSM)

The stained yolk–casein-mixture-based low-fat mayonnaises were stained with 1 mg/mL Nile red. Then, a confocal laser scanning microscope (CLSM) (Leica SP8, Weztlar, Germany) was used to observe the microstructure of the low-fat mayonnaise.

#### 2.2.6. Sodium Dodecyl Sulphate-Polyacrylamide Gel Electrophoresis (SDS-PAGE)

The 25 g yolk–casein-mixture-based low-fat mayonnaises with pre- and post-acid modification were centrifugated at 12,000 r for 30 min and collected the serum phase to obtain the proteins in the continuous phase. Then, the cream layer was mixed with 4% (*w*/*w*) Tween-20 in water (pH 4) to bring the total mass back to 25 g. After 24 h of continuous stirring, the samples were centrifugated at 12,000 r for 30 min and the serum phase was collected to obtain the desorbed proteins from the interface. Finally, the cream layer was mixed with isopropyl alcohol and centrifugated at 12,000 r for 30 min in triplicate. The serum phase was discarded to obtain the residual protein at the interface. All obtained proteins were diluted to 5 mg/mL for use in the SDS-PAGE analysis. The image of the gel was captured using MF-ChemiBIS 2.0 (DNR Bio-Imaging Systems Ltd., Jerusalem, Israel).

#### 2.2.7. Cryo-Scanning Electron Microscopy (Cryo-SEM)

The microstructure of all samples was observed by Cryo-SEM (SU8010, HITACHI, Tokyo, Japan). First, all samples were frozen in liquid nitrogen. Then, they were sublimed for about 20 min at −90 °C and coated with gold for about 60 s at 10 mA. Finally, the results were transferred to the cold stage to observe their structure. The cryo-SEM observation was conducted at 10 KV.

#### 2.2.8. Optical Microscopic

The morphology of all samples was observed by optical microscopic (Olympus, Tokyo, Japan). All samples were placed on a glass slide, and covered with a cover slide. Afterward, their morphologies were observed. Moreover, the particle size distribution of the emulsions was determined according to the method reported by Zhang et al. and Yu et al. [16,17]. Specifically, Image J software was applied to record and analyze the size of all emulsion droplets in micrographs by manually locating and segmenting the emulsion droplets.

#### 2.2.9. Sensory Evaluation

The low-fat mayonnaise samples were coded and put in plastic containers. Taking the commercially high-fat Kewpie mayonnaise as a reference, 20 semi-trained panelists assessed the low-fat mayonnaise samples from the perspectives of color, elasticity, viscosity, and acceptability using a 10-point scale (0 = vary greatly, 10 = little difference). All participants agreed to participate in the sensory evaluation experiment. Ethical permission was not required.

#### 2.2.10. Low-Field NMR (LF-NMR) Measurements

Approximately 1 g samples were placed in a bottle and then into the NMR coil. An LF-NMR analyzer (Suzhou Niumag Analytical Instrument Corporation, Suzhou, China) was employed with parameters as follows: Tw was 4500 ms, and the NECH was 6000. The relaxation time (T_2_) was obtained using the Carr Purcell Meiboom Gill sequence (CPMG).

#### 2.2.11. Rheological Measurements

A rheometer (TA Instruments, New Castle, USA) was used to assess the rheological properties of all samples. First, amplitude sweeps were carried out to identify the linear viscoelastic region (LVR). Subsequently, frequency sweeps were applied to ascertain the elastic modulus (G′ and G′’) of the samples in the LVR. Finally, the viscosity of all samples was determined at shear rates from 0.1 to 100 s^−1^.

#### 2.2.12. Statistical Analysis

All experiments were repeated at least three times. Statistical analyses were performed using SPSS Statistics 19 by Duncan multiple-range test and independent samples *T* Test (*p* < 0.05).

## 3. Results and Discussion

### 3.1. Interfacial Stability of Yolk–Casein-Mixture-Based Low-Fat Mayonnaise

**Figure 1:**
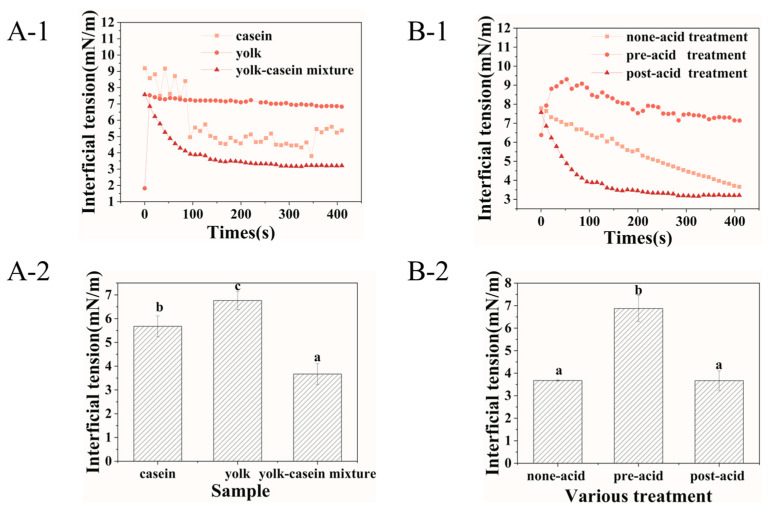
Interfacial tensions of casein, yolk–water with a mass ratio of 1:3, and yolk–casein mixture with a mass ratio of 1:3 with post-acid treatment (**A**) and the yolk–casein mixture with a mass ratio of 1:3 with various acid treatments (**B**). Figure (**A-2**) and (**B-2**) summarized interfacial tensions of samples according to Figure (**A-1**) and (**B-1**). Values with different letters (a–c) are significantly different (*p* < 0.05).

**Figure 2:**
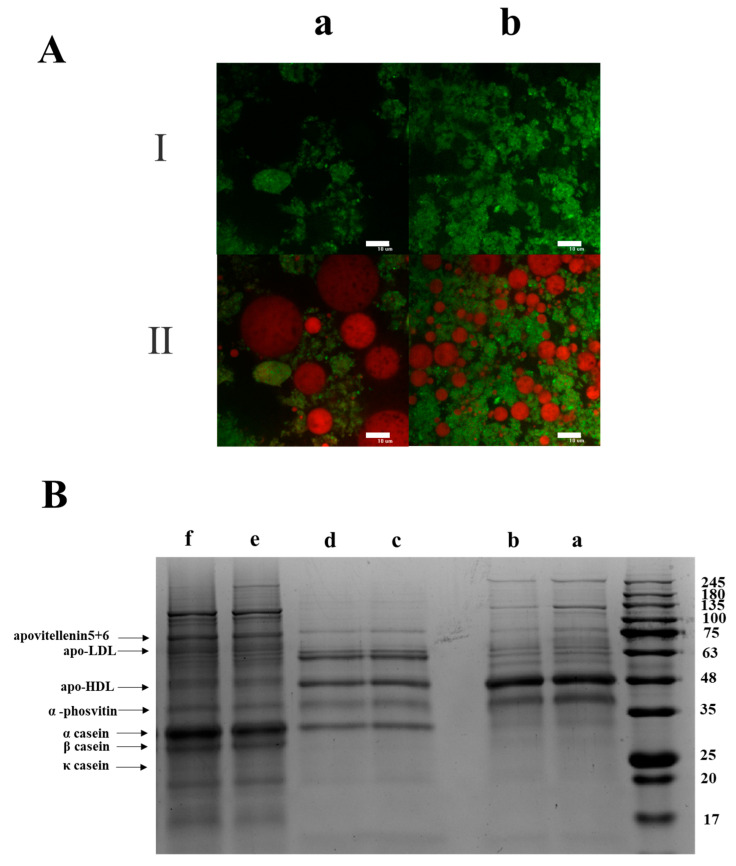
(**A**) CLSM images of stained yolk–casein-mixture-based low-fat mayonnaise with a mass ratio of 1:3 with pre-acid treatment (a) and post-acid treatment (b). The images containing single-channel images of FITC (green, for yolk) (I) and double-channel images of FITC (green, for yolk) and Nile red (red, for oil) (II). The scale bar is 10 μm. (**B**) SDS-PAGE of proteins in the continuous phase (a, b), desorbed proteins from interface (c, d), and residual protein at the interface (e, f) of pre-acid-treated (a, c, and e) and post-acid-treated (b, d, and f) yolk–casein-mixture-based low-fat mayonnaise with a mass ratio of 1:3.

Interfacial tensions were measured to assess the interfacial absorption behavior of casein, yolk, and yolk–casein mixture. As shown in Figure 1, all samples showed similar trends; that is, the interfacial tension decreased over time. The phenomenon could be attributed to the progressive adsorption of casein and the surface-active materials in the yolk at the oil–water interface [18]. Interestingly, under unified post-acidification conditions, it could be seen that the interfacial tension value of casein (5.68 ± 0.44 mN m^−1^) and yolk (6.76 ± 0.38 mN m^−1^) was always higher than those of the yolk–casein mixture (3.67 ± 0.43 mN m^−1^) (Figure 1(A-2)). These results indicated that casein and the surface-active materials in yolk synergistically decreased the interfacial tension, illustrating that the yolk–casein mixture was more conducive to the stability of emulsion than casein or yolk alone [19,20].

The acid sequence was an essential factor in affecting the interfacial absorption behavior of the yolk–casein mixture. As shown in Figure 1(B-2), the interfacial tension of the yolk–casein mixture with post-acid (3.67 ± 0.43 mN m^−1^) and non-acid (3.68 ± 0.02 mN m^−1^) treatment was lower than that with pre-acid treatment (6.84 ± 0.58 mN m^−1^). The decrease in the interfacial tension indicated the higher adsorption density of emulsifiers at the oil–water interface [21]. These results indicated that compared with pre-acid treatment, post-acid treatment was beneficial to the adsorption of casein and surface-active materials in yolk to the oil–water interface.

### 3.2. The Distribution of Yolk and Casein in Low-Fat Mayonnaise

The investigation of the distribution of yolk and casein was helpful in comprehending the stabilization mechanism of the yolk–casein-mixture-based low-fat mayonnaise. CLSM observations were carried out to obtain information about the chemical distribution of yolk in the emulsions. To perfectly analyze the contribution of yolk in the continuous phase and interface, the oil and yolk in the emulsion were dyed with Nile Red (red) and FITC (green), respectively. The casein protein was not stained. As shown in Figure 2A, the yolk and oil droplets in pre- and post-acid-treated low-fat mayonnaise showed a similar distribution. The green fluorescence was observed both on the surface of the oil droplets and in the continuous phase, and the green fluorescence was more concentrated in the continuous phase than at the oil–water interface. These results indicated that the distribution of yolk in the continuous phase was more than that at the interface. To study the contribution of casein in emulsions, SDS-PAGE was applied to separate and analyze the proteins in the continuous phase, desorbed proteins from the interface, and residual protein at the interface (Figure 2B). The casein bands were observed both in the continuous phase and the interface, and the casein bands separated from the residual protein at the interface were more obvious than those from the continuous phase and desorbed proteins at the interface. These results indicated that the distribution of casein at the interface was more than that in the continuous phase.

### 3.3. Post-Acid-Treated Yolk–Casein-Mixture-Based Emulsion to Form Low-Fat Mayonnaise

**Figure 3:**
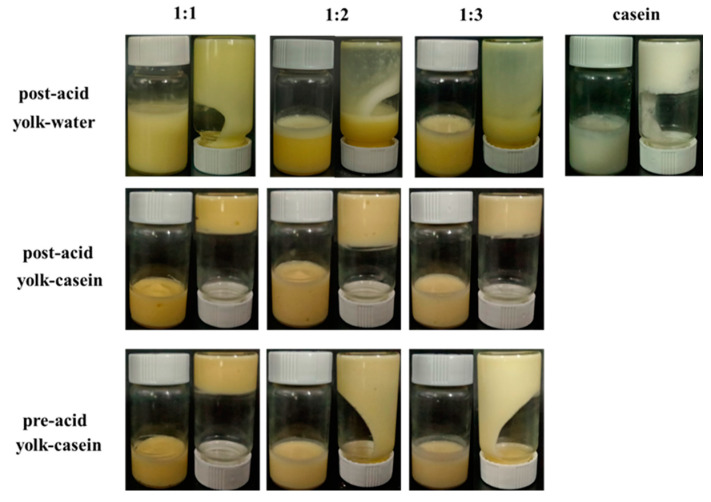
Visual appearances of the emulsions stabilized by casein, yolk–water with mass ratios of 1:1, 1:2, and 1:3 with post-acid treatment and the visual appearances of the low-fat mayonnaise prepared with yolk–casein mixture with mass ratios of 1:1, 1:2, and 1:3 with various acid treatment.

The visual appearances of the emulsions (30% oil content) using the casein, yolk, and yolk–casein mixture as stabilizers and treated with various sequences of acid are shown in Figure 3. The stabilizer was a crucial factor in the preparation of low-fat mayonnaise in this study. Under unified post-acidification conditions, the demulsification occurred immediately for the emulsions stabilized by casein, and the system with yolk–water had strong liquidity. Interestingly, yolk–casein-mixture-based low-fat mayonnaise was stable and showed excellent gel-like emulsion properties. These results verified that compared with casein or yolk alone, the yolk–casein mixture was an outstanding stabilizer for preparing semisolid low-fat mayonnaise.

Additionally, the acid sequence was another essential factor in influencing the preparation of the yolk–casein-mixture-based low-fat mayonnaise in this study. When the glass vials were inverted, the emulsion with pre-acid treatment flowed and could not attach to the glass vial well, while the low-fat mayonnaise with post-acid treatment displayed excellent semisolid behavior. In sensory evaluation, taking the commercial high-fat mayonnaise as a reference, the scores of all the low-fat mayonnaises with post-acid treatment were higher than the corresponding low-fat mayonnaises with pre-acid treatment in terms of color, elasticity, viscosity, and acceptability (Table 1). These results illustrated that the texture of yolk–casein-mixture-based low-fat mayonnaises with post-acid treatment was closer to that of commercial mayonnaise than the low-fat mayonnaises with pre-acid treatment.

### 3.4. The Dense Network Structure Formed by Self-Assembly of the Yolk and Casein “Traps” the Oil Droplets

**Figure 4:**
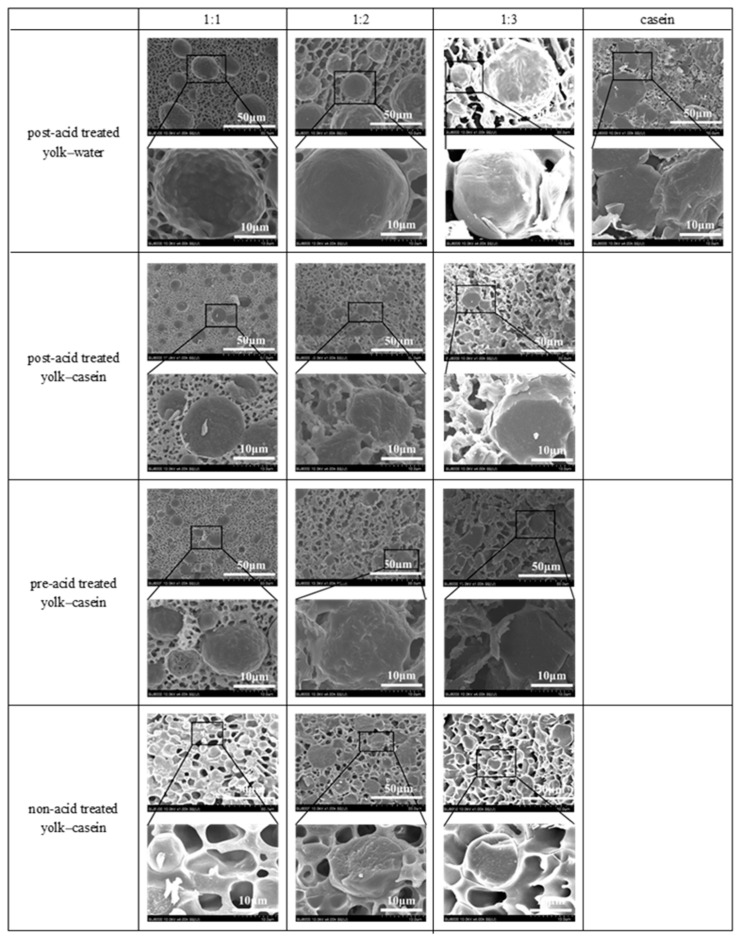
Cryo-SEM images of the low-fat emulsions stabilized by casein, yolk–water with mass ratios of 1:1, 1:2, and 1:3 with post-acid treatment and Cryo-SEM images of the low-fat mayonnaise prepared with yolk–casein mixture with mass ratios of 1:1, 1:2, and 1:3 with various acid treatments.

The cryo-SEM images of the low-fat mayonnaise are shown to illustrate the microstructure of all emulsions. As shown in Figure 4, unlike casein-based emulsions, a gel-like network structure was observed in the yolk–casein-mixture-based emulsion. The network formation in the yolk–casein-mixture-based low-fat mayonnaise can be attributed to the crosslinking of lipoproteins and casein. Additionally, it is reported that these network structures in the continuous phase can prevent the movement and coalescence of oil droplets [22], enhancing the stability of the emulsion [22], which is consistent with the improving stability of the emulsions observed in Figure 3. In addition, it is worth noting that regardless of the ratio of yolk to casein, the size of the emulsion droplets in the yolk–casein-mixture-based emulsions was smaller than that in the yolk–water-based emulsions. This result can be attributed to the fact that the addition of casein could cooperate with the surface-active materials in yolk to form a more stable interfacial layer at the oil–water interface, which was confirmed in Figure 1A, thus reducing the degree of flocculation of emulsion droplets and decreasing the aggregation of emulsion droplets [23].

**Figure 5:**
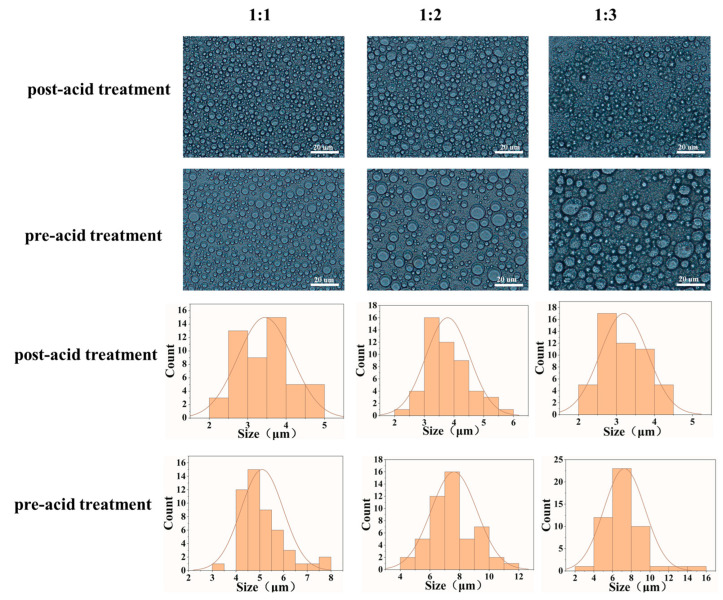
Optical microscopic images and droplet size distribution of low-fat mayonnaises prepared with yolk–casein mixture with mass ratios of 1:1, 1:2, and 1:3 and various acid treatments. The scale bar is 20 μm.

Moreover, we studied the influence of acid sequences on the microstructure of the yolk–casein-mixture-based emulsions. As shown in Figure 4, the structure of the network in the post-acid-treated low-fat emulsions was more compact and the pore size was smaller than that of non-acid-treated low-fat emulsions. These results indicated that the post-acid treatment induced the self-assembly of casein and lipoproteins in the continuous phase. Additionally, the low-fat emulsion with post-acid treatment showed a tighter network structure than that with pre-acid treatment. These results may be related to the emulsion droplet size and distribution in low-fat emulsions. The optical microscopic images and the statistical results of the emulsion droplet size distribution showed that regardless of the ratio of yolk to casein, compared with the fitted droplet size distribution curve of post-acid treated emulsion, the fitted droplet size distribution curve of pre-acid treated emulsion tended toward a larger particle size direction (Figure 5). This indicates that the size of emulsion droplets in the post-acid-treated low-fat emulsions was smaller than that of pre-acid-treated low-fat emulsions. These results can be attributed to the fact that compared with pre-acid-treated low-fat emulsions, the post-acid-treated low-fat emulsions had a more stable interfacial layer, which was confirmed in Figure 1B, thus decreasing the aggregation of emulsion droplets [23]. The aggregation of emulsion droplets led to a decrease in the interdroplet contact area and an increase in the unoccupied space between emulsion droplets [24]. Thus, compared with post-acid-treated low-fat emulsions, the network in pre-acid-treated low-fat emulsions was looser. The network structure in emulsions may influence their physical properties. A tight network structure could hinder the movement of the emulsion droplets in the emulsion system and enhance the gel properties well [25].

### 3.5. Enhancement Effect of Post-Acidification on Viscosity of Yolk–Casein-Mixture-Based Low-Fat Mayonnaise

**Figure 6:**
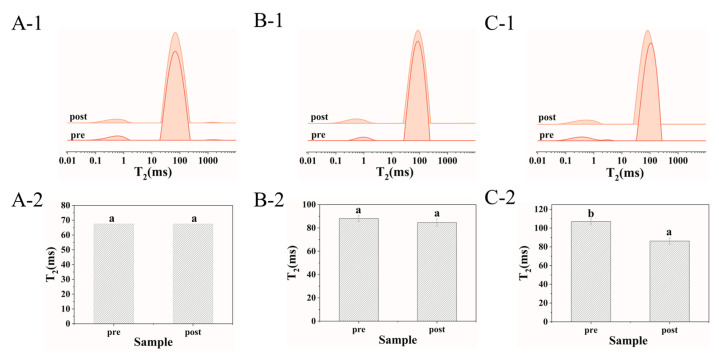
LF-NMR spectra of the low-fat mayonnaise prepared with yolk–casein mixture with mass ratios of 1:1 (**A**), 1:2 (**B**), and 1:3 (**C**) with pre-acid and post-acid treatment. Figure (**A-2**–**C-2**) summarized T_2_ values of samples according to Figure (**A-1**–**C-1**). Values with different letters (a–b) are significantly different (*p* < 0.05).

LF-NMR was used to evaluate the improvement in the physical properties of yolk–casein-mixture-based low-fat mayonnaise with post-acid treatment. T_2_ values can effectively provide information about the fluidity of water and oil droplets in the emulsion system [26,27]. As shown in Figure 6(A-1–C-1), all samples displayed two prominent peaks in the T_2_ spectra. Since the hydrogen protons in water relaxed more slowly than those in oil, the two peaks at around 0.1–1 ms and 10–1000 ms corresponded to the oil and water peaks, respectively [28]. The T_2_ value of the water peak was considered a suitable parameter to assess the viscosity of the emulsions [29]. It is well known that the relaxation time is positively correlated with water mobility; that is, the shorter the relaxation time, the lower the water mobility [26]. The viscosity of emulsions with lower T_2_ values was higher than that of emulsions with higher T_2_ values due to the decrease in molecular mobility [29,30]. According to the statistics, it could be clearly seen that the T_2_ values of the yolk–casein-mixture-based low-fat mayonnaise with post-acid treatment were lower than those with pre-acid treatment, especially for the low-fat mayonnaise prepared with yolk–casein mixture with a mass ratio of 1:3. This finding indicates that the viscosity of yolk–casein-complex-based low-fat mayonnaise with post-acid treatment was higher than that with pre-acid treatment.

### 3.6. Enhancement Effect of Post-Acidification on Rheological Properties of Yolk–Casein-Mixture-Based Low-Fat Mayonnaise

**Figure 7:**
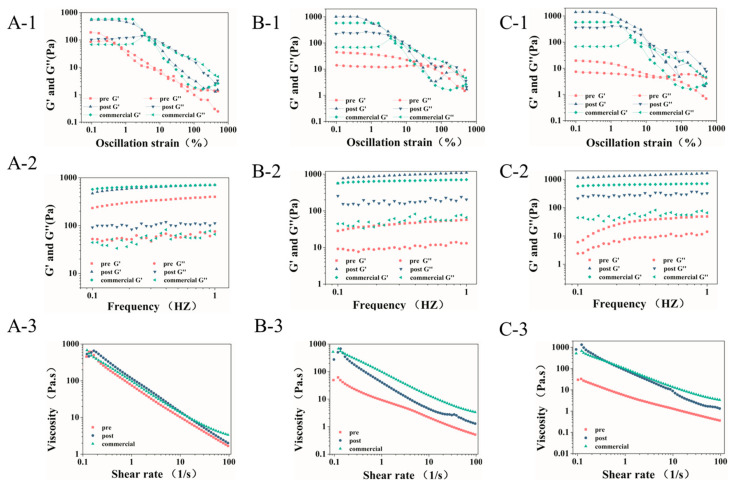
Rheological properties of the commercial mayonnaise and the low-fat mayonnaise prepared with yolk–casein mixture with mass ratios of 1:1 (**A**), 1:2 (**B**), and 1:3 (**C**) with pre-acid and post-acid treatment. (**1**) Amplitude sweeps, (**2**) Frequency sweeps, and (**3**) Viscosity tests.

To further intuitively evaluate the improvement in the physical properties of the yolk–casein-mixture-based low-fat mayonnaises with post-acid treatment, we studied the rheological properties of these samples with amplitude sweeps, frequency sweeps, and viscosity tests (Figure 7).

In the amplitude sweeps, the elastic modulus (G′) values were invariably higher than the viscosity modulus (G”) value for all emulsions in the linear viscoelastic region (LVR), regardless of the ratio of yolk to casein (Figure 7(A-1–C-1)), which indicated that all samples showed high elasticity in LVR [12]. With increasing stress, all emulsions showed similar trends; that is, the G′ were abruptly lower than G”. This result could be attributed to the structural rearrangements of the emulsion drops with high stress [12]. Furthermore, the G′ and G” of all low-fat mayonnaise with post-acid treatment were significantly higher than those of emulsion with pre-acid treatment in LVR. The values of G′ and G” illustrated that the low-fat mayonnaise with post-acid treatment had higher viscoelasticity compared with the emulsion with pre-acid treatment, which was attributed to the formation of the denser network observed in Figure 4. The trends in the frequency sweeps (Figure 7(A-2-C-2)) were consistent with the results in Figure 7(A-1–C-1), further confirming the above conclusions. Taking low-fat mayonnaise with a 1:1 ratio of yolk to casein as an example, when the frequency was 1 Hz, the G′ of the pre-acid-treated low-fat mayonnaise was 399.493 Pa. At the same frequency, the G′ of the post-acid-treated low-fat mayonnaise was 710.296 Pa. According to the statistics, the G′ of post-acid-treated low-fat mayonnaise improved by 77.80% compared with pre-acid-treated low-fat mayonnaise.

Figure 7(A-3–C-3) presents the viscosity of the emulsions as a function of the shear rate. It can be seen that the viscosity of all emulsions showed typical shear-thinning behavior; that is, the viscosity gradually decreased as the shear rate increased, regardless of the ratio of yolk to casein. This result could be associated with the destruction of the network structure or the disruption of the oil droplet clusters in the emulsions [31]. Additionally, it is worth mentioning that the viscosity of all low-fat mayonnaises with post-acid treatment was higher than that of emulsions with pre-acid treatment. Taking low-fat mayonnaise with a 1:1 ratio of yolk to casein as an example, when the shear rate was 0.1 s^−1^, the viscosity of pre-acid-treated low-fat mayonnaise was 230.041 Pa.s, while the viscosity of post-acid-treated low-fat mayonnaise was 437.488 Pa.s at the same shear rate. According to the statistics, the viscosity of post-acid-treated low-fat mayonnaise improved by 90.18%, compared with pre-acid-treated low-fat mayonnaise. These results can be attributed to the formation of a more robust network structure in the continuous phase induced by post-acid treatment, leading to the better restriction of the emulsion droplets in the low-fat mayonnaise system [25].

Moreover, the G′ and the viscosity of the low-fat mayonnaise with post-acid treatment were closer to that of commercial high-fat mayonnaise than the low-fat mayonnaise with pre-acid treatment regardless of the ratio of yolk to casein. These results indicated that compared with pre-acid treatment, the texture of yolk–casein-mixture-based low-fat mayonnaise with post-acid treatment was closer to that of commercially available high-fat mayonnaise, which was consistent with the sensory evaluation results.

### 3.7. Advantages of Post-Acidification Strategy for the Preparation of Low-Fat Mayonnaise

In general, protein-based fat replacers were widely applied in the preparation of low-fat mayonnaise with a similar texture to commercial high-fat mayonnaise. Table 2 summarizes the types and levels of ingredients used to create low-fat mayonnaise with protein-based fat replacers. For existing studies, the addition of chemically modified thickeners such as pectin, flaxseed gum, and xanthan gum was essential during the preparation of protein-based low-fat mayonnaise to increase the viscosity and reduce the fluidity of the products [2,3,8,32]. Compared with existing studies, the advantage of the post-acidification strategy for the preparation of low-fat mayonnaise applied here is that no thickeners are required. The developed clean-label thickener-free low-fat mayonnaise through post-acidification has the potential to attract more consumer attention.

## 4. Conclusions

This study provides a novel strategy for developing clean-label low-fat mayonnaise (30% oil content) via casein and yolk sequence self-assembly by post-acidification without additional thickeners. The results indicated that casein and yolk synergistically increased the stability of low-fat mayonnaise. For yolk–casein-mixture-based low-fat mayonnaise, a denser network structure with self-assembly of casein and yolk was formed in the low-fat mayonnaise with post-acid treatment than that with pre-acid treatment. The dense network achieved the viscoelasticity, viscosity, and semisolid properties required for low-fat mayonnaise, resulting in a good simulation of commercial high-fat mayonnaise in terms of texture.

## Figures and Tables

**Table 1:** Sensory evaluation of yolk–casein-mixture-based low-fat mayonnaises with pre-acid and post-acid treatment.

Sensory Parameters	1:1		1:2		1:3	
	Pre	Post	Pre	Post	Pre	Post
color	7.25 ± 1.11 ^a^	8.00 ± 0.82 ^b^	7.07 ± 1.43 ^a^	7.80 ± 1.01 ^a^	6.17 ± 0.93 ^a^	8.08 ± 0.10 ^b^
elasticity	7.22 ± 1.26 ^a^	8.65 ± 0.80 ^b^	5.82 ± 0.75 ^a^	8.15 ± 0.96 ^b^	6.30 ± 0.67 ^a^	7.67 ± 1.40 ^b^
viscosity	8.08 ± 1.03 ^a^	8.31 ± 1.18 ^a^	5.53 ± 0.97 ^a^	8.29 ± 0.61 ^b^	5.89 ± 0.78 ^a^	8.30 ± 0.70 ^b^
acceptability	7.77 ± 1.16 ^a^	8.66 ± 0.86 ^b^	5.22 ± 0.83 ^a^	7.63 ± 1.08 ^b^	5.18 ± 0.99 ^a^	8.09 ± 0.85 ^b^

Note: Data are presented as mean ± standard deviation. Values with different letters (a,b) are significantly different (*p* < 0.05).

**Table 2:** Processing ingredients (types and levels) in the preparation of low-fat mayonnaises.

Emulsifier	Protein-Based Fat Replacers	Thickeners	Fat Content	References
egg yolk powder–whey protein–gelatin	whey protein gelatin	Cold-pressed chia seed oil by-product gum	30%	[32]
egg yolk–amaranth protein isolate	amaranth protein isolate	xanthan gum guar gum	30%	[3]
egg yolk–microparticulated whey protein	microparticulated whey protein	pectin	16–48%	[2]
dried egg yolk–whey protein microparticles	whey protein microparticles	flaxseed gum	15%	[8]

## Data Availability

The original contributions presented in the study are included in the article, further inquiries can be directed to the corresponding author.
